# Case Report: Persistent response to combination therapy of pemigatinib, chemotherapy, and immune checkpoint inhibitor in a patient with advanced intrahepatic cholangiocarcinoma

**DOI:** 10.3389/fimmu.2023.1124482

**Published:** 2023-05-24

**Authors:** Zhuochao Zhang, Gaofei Wang, Lei Du, Jie Zhao, Lichao Pan, Gong Zhang, Fei Wang, Rong Liu

**Affiliations:** ^1^ Faculty of Hepato-Pancreato-Biliary Surgery, The First Medical Center of Chinese People’s Liberation Army (PLA) General Hospital, Beijing, China; ^2^ Key Laboratory of Digital Hepetobiliary Surgery, PLA, Beijing, China; ^3^ Department of Pathology, The First Medical Center of Chinese People’s Liberation Army (PLA) General Hospital, Beijing, China; ^4^ Department of Nuclear Medicine, The First Medical Center of Chinese People’s Liberation Army (PLA) General Hospital, Beijing, China; ^5^ Medical School of Chinese PLA, Beijing, China

**Keywords:** advanced intrahepatic cholangiocarcinoma, combined therapy, FGFR inhibitors, immune checkpoint inhibitor, pemigatinib

## Abstract

Patients with advanced intrahepatic cholangiocarcinoma (iCCA) often have a poor prognosis. Recent advancements in targeted molecular therapy and immunotherapy have been made. Herein, we report a case of advanced iCCA treated with a combination of pemigatinib (a selective FGFR inhibitor), chemotherapy, and an immune checkpoint inhibitor. A 34-year-old female was diagnosed with advanced iCCA with multiple liver masses and metastases in the peritoneum and lymph nodes. Next-generation sequencing (NGS) identified the genetic mutations. An FGFR2-BICC1 gene fusion was found in this patient. The patient was treated with pemigatinib in combination with pembrolizumab plus systemic gemcitabine and oxaliplatin. After 9 cycles of the combination therapy, the patient achieved a partial response, complete metabolic response, and normalization of tumor markers. Sequentially, the patient received pemigatinib and pembrolizumab for 3 months. Due to the elevated tumor biomarker, she is currently receiving chemotherapy, pemigatinib, and pembrolizumab treatment again. She regained an excellent physical status after 16 months of treatment. To the best of our knowledge, this was the first reported case of advanced iCCA successfully treated with a combination of pemigatinib, chemotherapy, and ICIs as a first-line regimen. This treatment combination may be effective and safe in the advanced iCCA.

## 1 Introduction

Cholangiocarcinoma (CCA) is categorized into anatomical subtypes, such as intrahepatic CCA (iCCA), perihilar CCA (pCCA), and distal CCA (dCCA) (1). The iCCA has received much attention in recent years due to its increasing global incidence (2). The intrahepatic mass lesion is its primary clinical manifestation. Radical surgery is primarily performed in patients with resectable cancer lesions (3). Patients with iCCA are often asymptomatic at the early stage of its pathogenesis, and it is mostly diagnosed at the advanced stage after the vascular invasion and distant metastasis. Patients in the advanced stage cannot undergo surgery and often have a poor prognosis. The median overall survival (OS) in untreated iCCA patients was 3 to 6 months (4).

Systemic therapies primarily using gemcitabine were the standard treatment during the past decades. Gemcitabine is combined with platinum-based compounds, and the combination therapy provides a median survival of fewer than 12 months (5). In recent years, immunotherapy brought encouraging results in treating several cancers. Many previous studies showed the advantage of combining immune checkpoint inhibitors (ICIs), tyrosine kinase inhibitors (TKIs), and chemotherapeutics for treating iCCA (6). National Medical Products Administration (NMPA) approved camrelizumab combined with chemotherapeutics (gemcitabine plus oxaliplatin) as a first-line systemic treatment for advanced biliary tract cancer (BTC) based on the outcomes of phase II clinical study (6). Moreover, novel targeted therapies, such as EGFR and VEGF inhibitors, IDH1/2 inhibitors, and FGFR inhibitors were tried in recent years. Genomic alterations in FGFR were found in multiple cancer types. The occurrence of FGFR2 alterations in cholangiocarcinoma, especially in iCCA, ranged from 10% to 15% (7). Recently, pemigatinib, a selective FGFR1-3 inhibitor, was approved by the US FDA and China NMPA for the treatment of unresectable locally advanced or metastatic CCA with an FGFR2 fusion or other rearrangements for adults who had previous other treatments. A combination of immunotherapy, chemotherapy, and TKI was used in the treatment of iCCA, and the preliminary data showed better benefits using this combination (8). However, the efficacy and safety of this combination of ICI, chemotherapeutic, and FGFR inhibitor against iCCA with altered FGFR2 gene remain unclear.

Herein, we report a case of advanced iCCA treated with a combination of PD-1 antibody, chemotherapy, and pemigatinib that demonstrated efficacy and safety. Encouragingly, this patient achieved a metabolic complete response. To the best of our knowledge, this is the first case treated with this combination therapy.

## 2 Case presentation

A 34-year-old female patient was admitted to our hospital in October 2021 with the complaints of right upper abdominal pain and lower abdominal distension. On physical examination, the left cervical lymph node was enlarged to the size of 2x2 cm^2^, with tenderness and undefined boundaries. Multiple liver masses with a maximal tumor diameter of 57 mm and invasion of the inferior vena cava and the left branch of the portal vein were revealed by contrast-enhanced abdominal and pelvic magnetic resonance imaging (MRI). Multiple nodules with various enhancements were present in the perihepatic peritoneum, omentum, and ovary. The peritoneum was thickened in the area of the Douglas fossa. A small amount of ascitic fluid was found on the ultrasonographic image. Positron emission tomography/computed tomography (PET/CT) scans displayed multiple liver masses with increased glucose metabolism, and other sites, such as the ovaries, perihepatic peritoneum, omentum, liver portal lymph node, and retroperitoneal nodes. The left cervical lymph node had masses with high SUVs. The patient had the Eastern Cooperative Oncology Group score of 1. The serum cancer antigen 125 (CA125) significantly increased to 358 µ/mL (normal reference range: 0.1-35 µ/mL). Other tumor biomarkers, such as alpha-fetoprotein (AFP), carcinoembryonic antigen (CEA), cancer antigen 724 (CA724), and cancer antigen 19-9 (CA19-9) levels, were within the normal range.

**Figure 1:**
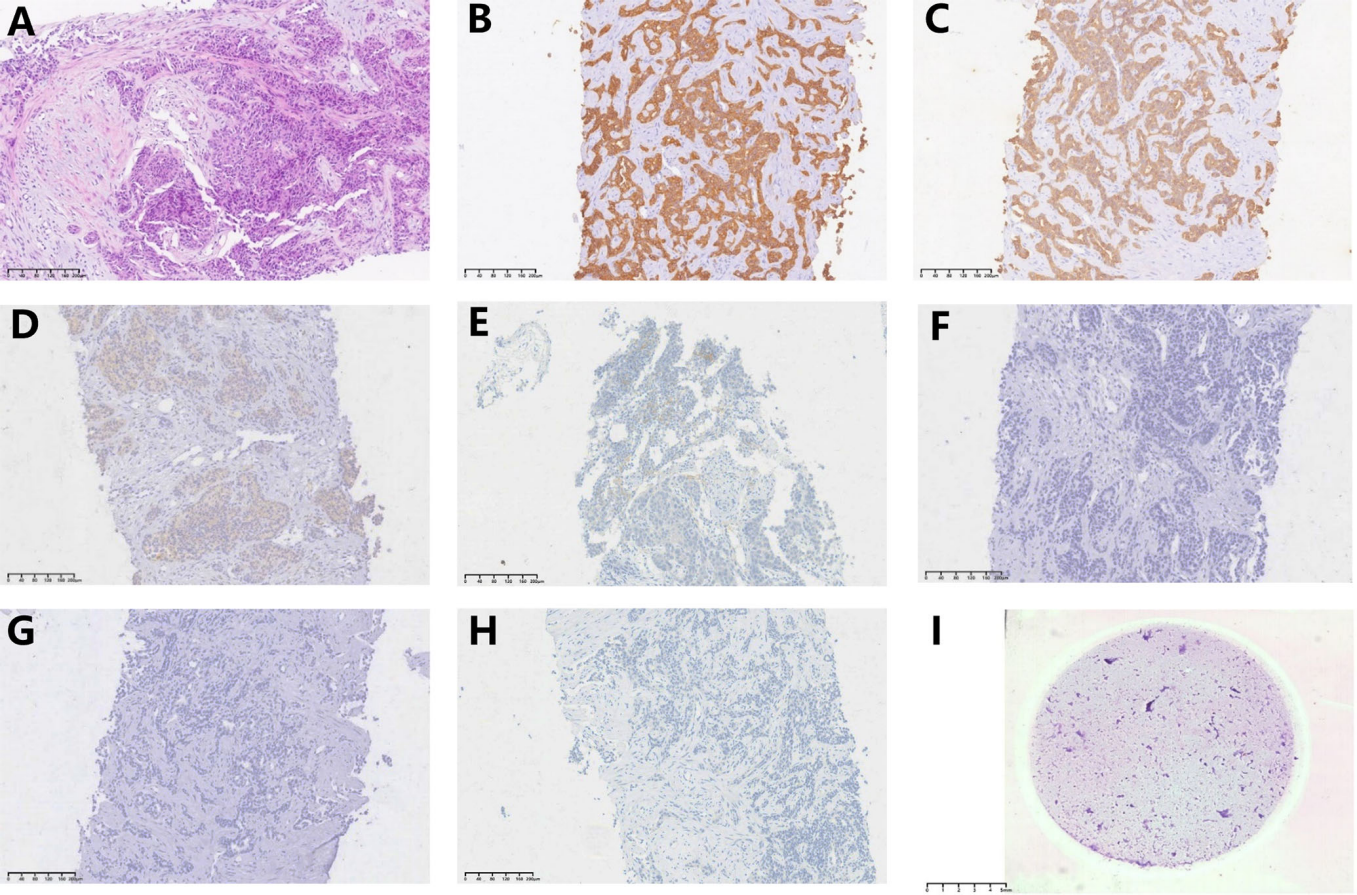
Hematoxylin-eosin (H&E) staining and immunohistochemical evaluation of the tissue obtained using needle biopsy (10x). Pathological images show **(A)** (H&E) staining, **(B)** positive staining for CK18, **(C)** positive staining for CK19, **(D)** positive staining for PD-1, **(E)** positive staining for PD-L1(10% positive), **(F)** negative staining for CA125, **(G)** negative staining for hepatocytes, **(H)** negative sating for ER, and **(I)** H&E staining of tumor cells from the ascitic fluid (0.5x).

**Figure 2:**
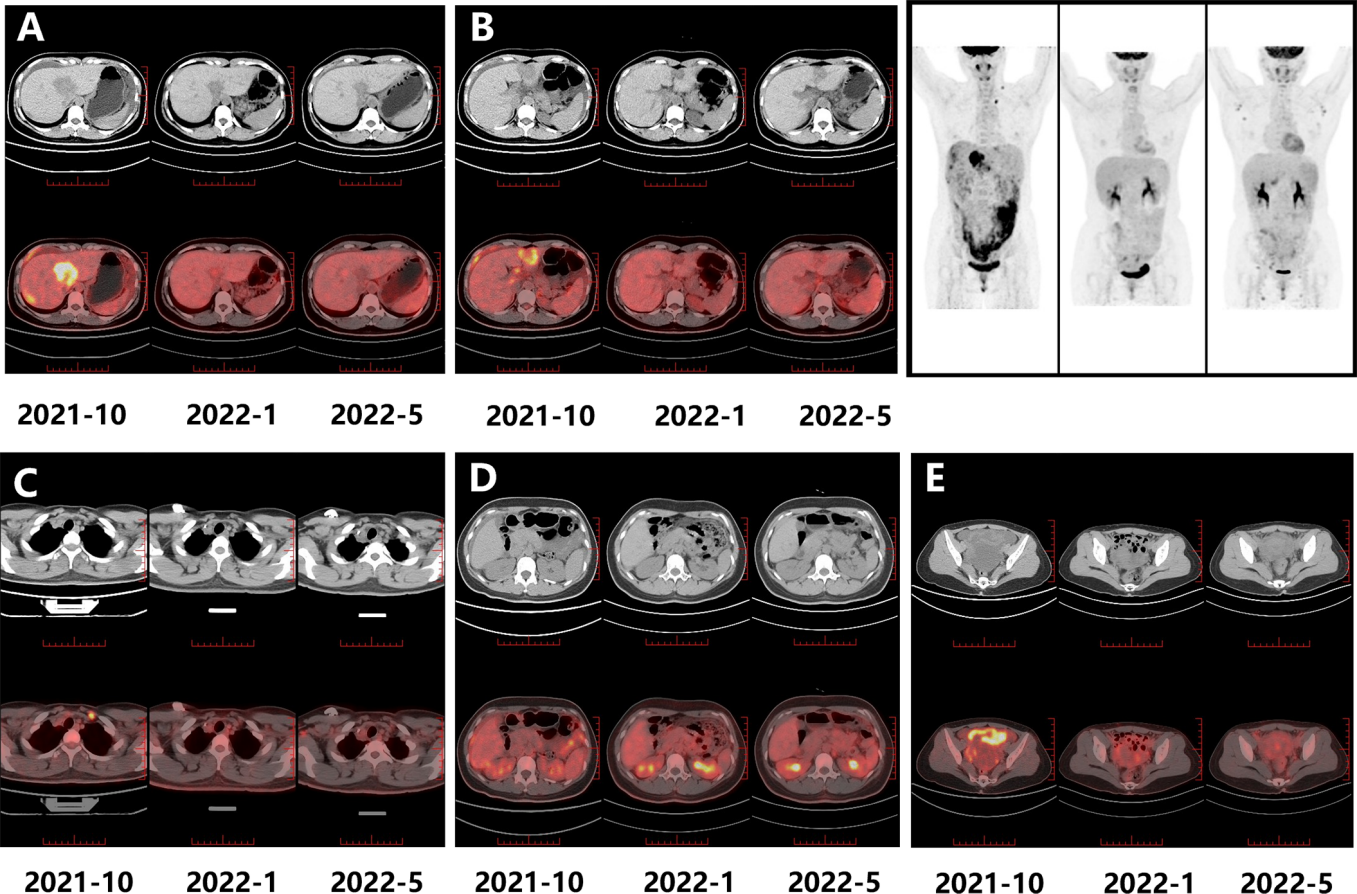
The PET-CT images for evaluating the treatment response. The metabolic activity change is noticeable in the masses located in the right liver **(A)**, left liver **(B)**, left cervical lymph node **(C)**, retroperitoneal nodes **(D)**, and omentum and ovary **(E)** (2021-10 is the baseline).

**Figure 3:**
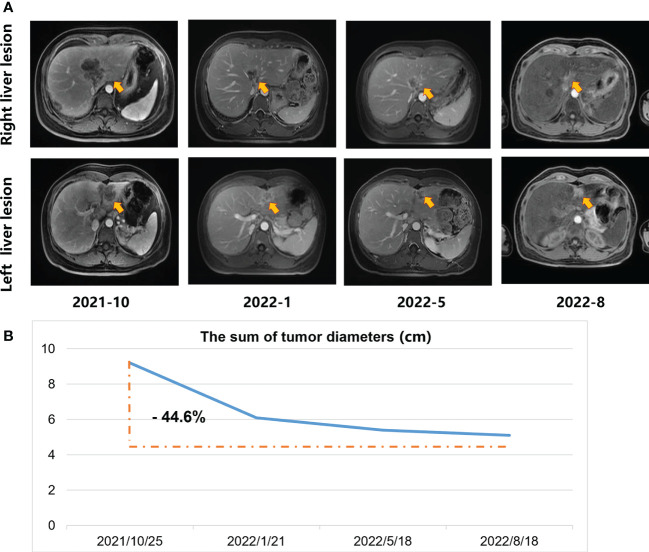
The MRI scans during the systemic treatment. **(A)** Upper-row images indicate the maximum diameter of the lesion located in the right liver; Lower row images denote the maximum diameter of the lesion located in the left liver. Arrows in the figures indicate the position of the lesions. **(B)** The sum of tumor diameters.

**Figure 4:**
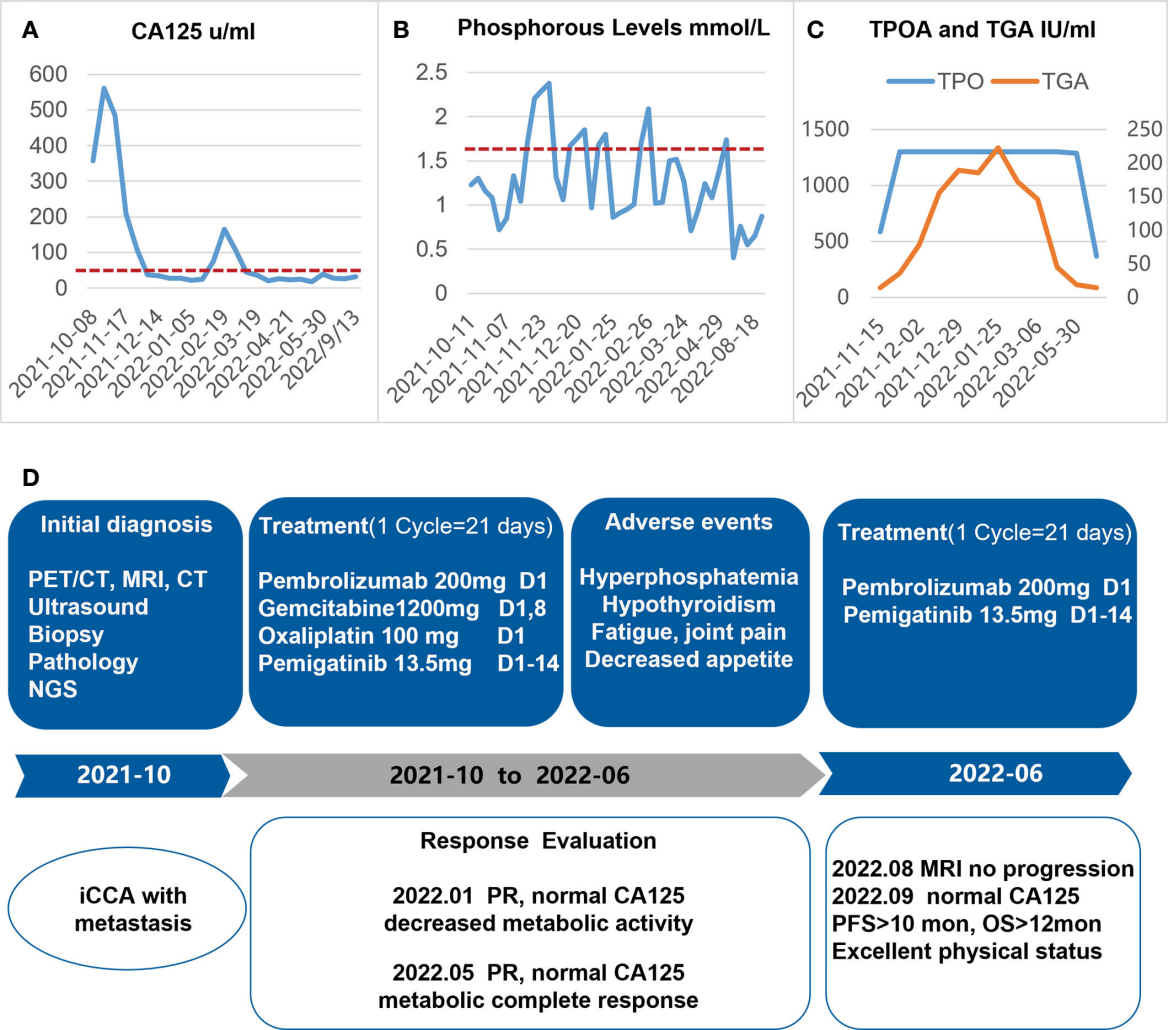
Diagram depicting the dynamic changes of CA125 **(A)**, phosphorous level **(B)**, and TPOA/TGA **(C)** over time, after the onset of systemic therapy. **(D)** The timeline of the treatment protocol. iCCA, intrahepatic cholangiocarcinoma; NGS, next-generation sequencing; PR, partial response.

An ultrasound-guided needle biopsy was performed on October 14, 2021, for the pathological evaluation of the tumor mass. The pathological diagnosis was moderate to poorly differentiated adenocarcinoma (**Figure 1A**). On immunohistochemical evaluation, the tumor tissue was positive for cytokeratin 18, cytokeratin 19, PD-1, and PD-L1 (10%) (**Figures 1B–E**) and negative for CA125, hepatocytes, and ER (**Figures 1F–H**). The patient was diagnosed with advanced intrahepatic cholangiocarcinoma with the grade of T2N1M1, according to the American Joint Committee on Cancer (AJCC) 8th edition guidelines. Next-generation sequencing was performed using biopsy samples. An FGFR2-BICC1 gene fusion was found in this patient. It is the most common pattern of FGFR2 fusion in the iCCA (9). The detailed fusion pattern of this patient was FGFR2: exon17-BICC1: exon 3 and FGFR2: exon17-del47ins121-BICC: exon 2, with an abundance of 39822 copies. The health of the patient deteriorated rapidly with severe lower abdominal distension. Moderate to severe ascites was detected by ultrasonography. Tumor cells were identified in the ascitic fluid (**Figure 1I**). The serum CA125 significantly increased to 560.9 µ/mL from 358 µ/mL within two weeks of the admission. Pembrolizumab combined with chemotherapeutics (gemcitabine plus oxaliplatin (gemox)) and pemigatinib were administered. Pembrolizumab (200 mg on d1), gemcitabine (800 mg/m^2^ on d1 and d8), oxaliplatin (65 mg/m^2^ on d1), and pemigatinib (13.5 mg/d on d1-d14) were administered for 1 cycle of 21 days. During the therapeutic course, the MRI and PET/CT were performed every 3-4 months. After 3 months of treatment, an MRI examination in January 2022 revealed a partial response, and the sum of tumor diameters was reduced by 39.1%. The PET/CT images displayed a significant decrease in tumor size and metabolic activity. The serum CA125 level decreased to the normal range in December 2021. A treatment interruption, due to personal reasons, led to a rapid rebound of CA125 to 165.2 µ/mL in February 2022. However, the level came to normal after the resumption of treatment. After 7 months of therapy, assessments were completed in May 2022. On MRI examination, the sum of tumor diameters was reduced by 57.2% of the baseline value. The PET/CT scans showed achievement of tumor metabolic activity complete response (**Figure 2**). The serum CA125 level remained normal. After the discussion by a multidisciplinary team (MDT) and considering the patient’s tolerance, the therapy scheme was adjusted to use PD-1 antibody and pemigatinib, q3w. After 3 treatment cycles, the serum CA125 level was still in the normal range. An MRI examination in August 2022 revealed no progression, compared to the finding in May 2022. Compared to the baseline level, the sum of tumor diameters was reduced by 44.6% (**Figure 3**). However, the serum CA125 increased to 142.9 µ/mL in November 2022. On an abdominal MRI in November 2022, abdominal lymph nodes were enlarged although the change of the lesion located in the liver was not obvious. The patient was switched to the previous treatment, but a severe allergic reaction occurred during the infusion of oxaliplatin. Thus, the therapy scheme was adjusted to use nab-paclitaxel, S-1, PD-1 antibody, and pemigatinib. The serum CA125 decreased to 61.83 µ/mL after 1 cycle of the therapy (**Figure 4A**). The last follow-up date was on February 19, 2023. Due to the infection of COVID-19 in January 2023, her treatment was interrupted. She had a PFS of more than 12 months and OS of more than 16 months after receiving the combination therapy. The timeline of treatment is shown in **Figure 4D**.

The adverse reactions to this combination therapy were observed. The main treatment-related toxicities were hyperphosphatemia, hypothyroidism, fatigue, decreased appetite, and joint pain. Intake of sevelamer carbonate (0.8 g, tid) decreased the blood phosphorus levels during pemigatinib administration (**Figure 4B**). The patient was diagnosed with Hashimoto’s thyroiditis 2 years ago when she was pregnant. During this treatment procedure, the patient had hypothyroidism and diffuse hyperplasia of the thyroid gland. Significant elevation of TPOA (anti-thyroid peroxidase antibody) and TGA (anti-thyroglobulin antibody) was found, which may have been due to the administration of the PD-1 antibody. After receiving levothyroxine replacement, the abnormal TPOA and TGA gradually returned to near-normal levels (**Figure 4C**), and her symptoms eased at the same time. Very mild myelosuppression was noticed. Overall, the adverse reactions related to this treatment were mild, and no adverse reactions were more severe than the grade 2.

This patient felt miserable when she was diagnosed with metastatic iCCA. She consented to the treatment scheme. After receiving the combined therapy, she noticed a fast improvement in her condition. She was relieved when she was told her tumor had no metabolic activity and the serum CA125 was within normal range. She gladly agreed to reveal her case.

## 3 Discussion

A combination therapy consisting of pemigatinib, chemotherapy, and ICI as a first-line treatment regimen in advanced ICCA with FGFR2 fusion may be effective and safe.

Most iCCAs are diagnosed in the advanced stage of the disease. Systemic chemotherapy is the first option for the advanced stage of iCCA (10). However, only a few studies on chemotherapy exist for the treatment of iCCA. Currently, chemotherapy using cisplatin and gemcitabine is used as the standard therapy, based on the outcome from the ABC-02 phase C-clinical study on patients with BTC (5). Other combination therapies, including the combination of oxaliplatin, irinotecan, and infusible fluorouracil (mFOLFIRINOX), gemcitabine plus oxaliplatin (GEMOX), and cisplatin, gemcitabine plus S-1, gemcitabine plus nab-paclitaxel, and gemcitabine, cisplatin, and nab-paclitaxel also showed comparable outcomes (11–15). Change of chemotherapy schemes did not appear to further improve therapeutic efficacy. Recently, an increasing number of studies reported that immune checkpoint inhibitors when combined with chemotherapeutics showed promising outcomes with high response rates. Notably, data from the randomized phase III TOPAZ-1 trial were reported at the 2022 ASCO GI cancer symposium that durvalumab combined with gemcitabine plus cisplatin (GemCis) treatment significantly improved the outcome compared to GemCis treatment alone as a first-line therapy in advanced BTC. The combined therapy of first-line GemCis and durvalumab achieved a better OS (12.8 vs. 11.5 months), PFS (7.2 vs. 5.7 months), and ORR (26.7% vs. 18.7%) compared to those with GemCis (16). Recently, several studies evaluated the efficacy of combinations of antiangiogenic drugs and ICIs. However, the ORRs were inconsistent. Atezolizumab with or without bevacizumab in combination with cisplatin plus gemcitabine in phase II study IMBRAVE 151 in patients with untreated, advanced BTC was used (17). No significant difference in ORR was found between the two groups. However, the duration of response (DOR) of over 6 months was 89% for atezo plus bev plus CisGem vs. 47% for atezo plus placebo plus CisGem. The aggregate of data suggested that combining atezo with bev and chemotherapy may provide clinical benefit in a subset of patients with advanced BTC. The participants are being followed-up for determining the OS. Pembrolizumab was assessed when combined with lenvatinib through the phase II study LEAP-005 consisting of 31 patients with CCA who were treated previously with other treatments. The ORR was 10%, and the median PFS and OS were 6.1 months and 8.6 months, respectively (18). Zhang et al. reported that lenvatinib plus PD-1 inhibitors showed promising anti-tumor efficacy in patients with BTC. The ORR was 42.1% and the disease control rate (DCR) was 76.3% (19). These two studies had the limitation of small sample sizes. Larger studies with longer follow-ups are required to substantiate these findings. Interestingly, Zhou et al. reported that the combination of PD-1 antibody, GEMOX, and lenvatinib as the first-line therapy achieved an ORR of 80% and a PFS of 10 months. Therefore, combination therapy appears to have better outcomes (8).

With the clinical application of next-generation sequencing, several critical gene alterations associated with CCA were identified to pave ways for targeted precision therapies, focusing fibroblast growth factor receptor (FGFR), epidermal growth factor receptor (EGFR), human epidermal growth factor receptor 2 (HER2), isocitrate dehydrogenase 1 and 2 (IDH1/2), BRAF, and tyrosine kinase receptors (20, 21). Several targeted therapies have been elucidated *via* clinical trials in the last three years. Fibroblast growth factor (FGF) signaling regulates cell development and angiogenesis; FGFRs comprise a family of receptor tyrosine kinases (FGFR1-4). FGFR2 fusion is the most common genetic alteration identified in the iCCA, with the most common fusion partner candidate being BICC1 (22).

Pemigatinib was the first FGFR inhibitor approved by the US FDA and the China NMPA in April 2022 for use in patients with iCCA harboring FGFR2 fusions or rearrangements. This orally administered FGFR1-3 inhibitor demonstrated efficacy in the phase II FIGHT-202 study (23). In this clinical study, patients with FGFR fusions or rearrangements showed an objective response rate of 35.5%. Several other FGFR inhibitors, including infigratinib, erdafitinib, and futibatinib (TAS-120) were also used in the clinical practice with comparable outcomes, for example, the median duration of response was 9.7 months in the futibatinib (TAS-120) group (22). However, all these FGFR inhibitors were administered as the second-line therapy in patients with unresectable locally advanced or metastatic cholangiocarcinoma with an FGFR2 fusion or rearrangement who were previously treated. Currently, randomized phase III clinical studies are being conducted to compare pemigatinib or infigratinib with gemcitabine/cisplatin as the first-line therapy in patients with FGFR gene fusions or rearrangements (NCT03656536 and NCT03773302).

Whether the combination of FGFR2 inhibitors and other drugs in treating iCCA could be superior to monotherapy is still unanswered. Evidence from preclinical research suggested that a combination of FGFR inhibition and PD-1 suppression expanded the T-cell clones and caused immunological changes in the tumor microenvironment to enhance anti-tumor immunity and survival (24). As known, lenvatinib is a receptor tyrosine kinase inhibitor that targets bothVEGFR and FGFR. Some preclinical studies revealed the novel roles of FGFR signaling in regulating cancer immunity by inhibiting the IFNF pathway. The inhibitory activity of lenvatinib against FGFRs likely contributes to enhanced anti-tumor activity (25). This concept may have been one of the reasons that the combination of PD-1 antibody, GEMOX, and lenvatinib could have been more effective in Zhou et al.’s study. However, the information on the efficacy and safety of combination therapy containing ICIs, chemotherapy, and FGFR inhibitors for iCCA is presently lacking. Some clinical trials, such as the trial NCT05004974 are in progress to elucidate the efficacy and safety of combined therapy of ICI and FGFR inhibitor in non-small cell lung cancer.

With the experience of using different combinations of targeted therapeutics and chemotherapeutics, a framework of multi-dimensional timely treatment for malignant tumors was proposed through decision-making and response evaluation (26). The success of the combination drug strategy may depend on a metronomic low-dose treatment approach by reducing the dose of therapeutic agents using a highly efficient systemic management paradigm and expertise from the multidisciplinary team. Guided by this perception, we observed better outcomes with this combinatory treatment.

To the best of our knowledge, this was the first reported patient with metastatic iCCA treated with a combination of an FGFR inhibitor, an ICI, and chemotherapy. The patient achieved a sustained comparatively better response with a PFS of more than 12 months and an OS of more than 16 months, which were longer than most reports regarding advanced iCCA. The adverse effects were mild in this patient. The patient was currently on pemigatinib and pembrolizumab. She regained an excellent physical status after 12 months of treatment. Accumulation of additional cases and evidence from prospective studies involving advanced iCCA, especially metastatic iCCA, are required to validate the safety and efficacy of this treatment combination.

## Data availability statement

The original contributions presented in the study are included in the article/supplementary material. Further inquiries can be directed to the corresponding authors.

## Ethics statement

The studies involving human participants were reviewed and approved by The Ethics Committee of PLA General Hospital. The patients/participants provided their written informed consent to participate in this study. Written informed consent was obtained from the participant for the publication of this case report.

## Author contributions

Drafting of the manuscript: ZZ; Planning treatment decisions: FW and RL; Evaluating Imaging outcome: LD; Pathological interpretation: GW; Clinical data analysis and taking care of the patient: ZZ, JZ, LP, and GZ. All authors contributed to the article and approved the submitted version.
